# Functional Nanomaterials for Sensing Devices: Synthesis, Characterisation and Applications (2nd Edition)

**DOI:** 10.3390/nano16130833

**Published:** 2026-07-07

**Authors:** Barbara Vercelli

**Affiliations:** Istituto di Chimica della Materia Condensata e di Tecnologie per l’Energia, CNR-ICMATE, Via Cozzi, 53, 20125 Milano, Italy; barbara.vercelli@cnr.it

## 1. Introduction

In the current era, nanomaterials (NMs) are at the forefront of sensor development for a wide range of high-performance applications, including environmental monitoring, manufacturing processes and medical diagnosis [1,2]. Their unique properties, such as high surface area, tunable surface structures and advanced optical, electrical and mechanical features, can be exploited to easily engineer and fine-tune sensor configurations based on recognition phenomena occurring at the nanoscale level [3,4]. In particular, depending on the NM characteristics and the specific target, a plethora of analytical techniques, ranging from electrochemical detection to optical, fluorescence, surface plasmon resonance, and colorimetric methods, can be employed in the design of specific sensing devices [5,6,7,8,9].

Building on these considerations, this Special Issue (SI) provides an overview of the advances, challenges and opportunities associated with the application of NMs in sensing technologies. Due to the success of the previous issue, this Special Issue represents its second edition. It focuses particularly on the synthesis and application of NMs such as silver-based nanoclusters [10,11], carbon-based nanostructures [12,13] and electrically responsive artificial muscles [14] in sensing devices and their various principles and mechanisms, including surface-enhanced Raman spectroscopy (SERS), electrical stimulation, and fluorescent and colorimetric responses.

The aim of the collected contributions is to provide interested scholars with a ‘toolbox’ on the synthesis, characterisation, and use of NMs in sensing technologies, as detailed in the following section.

## 2. An Overview of the Published Articles

The SI includes several interesting contributions on silver-based nanoclusters. In particular, M. Cuccioloni et al. [10] report the development of a colorimetric sensor based on silver nanoparticles functionalised with mercaptoundecanoic acid for determining the presence of heavy metals Cd^2+^, Cu^2+^, Mn^2+^, Ni^2+^ and Zn^2+^ in water. Conversely, Ketkong A. et al. [11] prepared triangular silver nanostructures with sharp corners for use as active materials in surface-enhanced Raman spectroscopy (SERS) for the qualitative and quantitative detection of paraquat in water samples simulating natural contamination.

Other fascinating contributions deal with the use of sustainable, luminescent, carbon-based nanomaterials as a substitute for classical quantum dots based on critical raw materials (CRMs) in sensing applications. Nguyen D. H. H. et al. [12] describe the sustainable synthesis of carbon quantum dots (CDs) from the Maillard reaction between glucose and glycine and their use as fluorescent probes for the selective detection of elemental selenium. Vercelli B. et al. [13] discuss the nature of the interactions between CDs and promising food preservatives such as pyrogallol, benzoic acid and gallic acid. This work is framed within the design of CD-based sensors capable of determining safe levels of these compounds in food and beverages, as well as the development of sustainable, intelligent packaging systems for controlling food shelf life.

Another notable contribution reports on the development of electrically responsive artificial muscles. Salim N. V. et al. [14] investigated the impact of treating liquid crystalline elastomers (LCEs) with polydopamine (PDA) prior to functionalisation with an electrically conductive gold-sputtered coating. They discovered that this treatment significantly enhanced the adhesion of the gold coating to the LCEs, resulting in the creation of LCEs with multifunctional, strain-sensing, electrically conductive and electro-responsive properties.

## 3. Conclusions

In conclusion, the contributions to this second edition of the SI reflect the widespread interest in the design, synthesis, characterisation and potential sensing applications of functional nanostructured materials. While this SI cannot cover all aspects of functional nanostructured materials, the contributions presented herein provide insights into fundamental research, fostering development and innovation and improving the application of these materials in technological fields.
